# Epigenetic Regulation of Cancer-Associated Genes in Ovarian Cancer

**DOI:** 10.3390/ijms12020983

**Published:** 2011-01-31

**Authors:** Mi Jeong Kwon, Young Kee Shin

**Affiliations:** 1 Advanced Institutes of Convergence Technology, Suwon, Kyeonggi-do 443-270, Korea; E-Mail: mjkwon94@snu.ac.kr; 2 Department of Pharmacy, College of Pharmacy, Seoul National University, Seoul 151-742, Korea

**Keywords:** ovarian cancer, epigenetic derepression, DNA methylation, histone modification, miRNA, epigenetic therapy, chemoresistance, cancer-initiating cells

## Abstract

The involvement of epigenetic aberrations in the development and progression of tumors is now well established. However, most studies have focused on the epigenetic inactivation of tumor suppressor genes during tumorigenesis and little is known about the epigenetic activation of cancer-associated genes, except for the DNA hypomethylation of some genes. Recently, we reported that the overexpression of cancer-promoting genes in ovarian cancer is associated with the loss of repressive histone modifications. This discovery suggested that epigenetic derepression may contribute to ovarian tumorigenesis by constituting a possible mechanism for the overexpression of oncogenes or cancer-promoting genes in tumors. The emerging importance of epigenetic aberrations in tumor initiation and in the regulation of cancer-initiating cells, suggests that epigenetically regulated genes may be promising therapeutic targets and biomarkers. Given that the current challenges in ovarian cancer include the identification of biomarkers for early cancer detection and the discovery of novel therapeutic targets for patients with recurrent malignancies undergoing chemotherapy, understanding the epigenetic changes that occur in ovarian cancer is crucial. This review looks at epigenetic mechanisms involved in the regulation of cancer-associated genes, including the contribution of epigenetic derepression to the activation of cancer-associated genes in ovarian cancer. In addition, possible epigenetic therapies targeting epigenetically dysregulated genes are discussed. A better understanding of the epigenetic changes in ovarian cancer will contribute to the improvement of patient outcomes.

## 1. Introduction

Ovarian cancer is the most lethal gynecological malignancy [1]. Due to difficulties in early detection, most ovarian cancers are diagnosed at an advanced stage. Furthermore, most patients diagnosed at an advanced stage show a poor prognosis because recurrence occurs in the majority of patients, although most respond to current standard chemotherapies [2]. Therefore, a better understanding of the molecular pathogenesis of ovarian cancer is required so that novel biomarkers facilitating early detection and the development of new therapeutic targets are identified.

Ovarian cancers are very heterogeneous at the clinical, histopathological and molecular levels [3]. More than 90% of ovarian cancers are epithelial in origin and are thought to arise from the ovarian surface epithelium (OSE) or from surface epithelial inclusion cysts [3,4]. As is well known in other forms of human tumorigenesis, epithelial ovarian cancers are caused by both multiple genetic and epigenetic alterations [4]. They develop due to the accumulation of genetic changes in multiple oncogenes and tumor suppressors. These genetic changes drive alterations in cellular signaling pathways that are some of the hallmarks of cancer and contribute to ovarian tumorigenesis [4]. To date, more than 15 oncogenes and 16 putative tumor suppressor genes have been implicated in ovarian oncogenesis [4]. The most well-known tumor suppressor genes that are frequently lost or inactivated in epithelial ovarian cancer include *TP53*, *PTEN*, *BRCA1* and *BRCA2*. Commonly activated oncogenes are *KRAS*, *BRAF*, and *PIK3CA*. In addition, several signaling pathways are frequently activated in ovarian cancer, including the Ras-MAPK signaling pathway, the PI3K pathway, IL6-IL6R-Jak2-STAT3 signaling, and the LPA and NF-kB signaling pathways, amongst others [2,4]. Based on these genetic aberrations, several potential anti-cancer targets have been identified recently and novel targeted therapeutics are under development [2,5]. The main focus of current targeted therapies is the inhibition of the oncogenes or signaling pathways that are most frequently activated or overexpressed in ovarian cancer and, which, it is assumed, provide synthetic lethality. However, given that the prevention of tumor recurrence after therapy is one of the main challenges in the treatment of ovarian cancer, and that cancer-initiating cells (CICs) may be involved in drug resistance, the development of novel molecules or approaches targeting CICs is very important. Recent studies have proposed that epigenetic changes play a central role during the early stages of tumorigenesis and in the regulation of CICs [6,7]. Moreover, it is generally held that the activation of oncogenes occurs through genetic changes, including amplification and mutation, whereas tumor suppressor genes are inactivated by events such as promoter methylation, gene deletion and loss of heterozygosity (LOH). However, genetic changes such as gene amplification and mutation alone are insufficient to explain the activation of oncogenes or cancer-promoting genes in ovarian cancer, and the possible role of an epigenetic mechanism in the activation of cancer-associated genes has been suggested recently, although it is little understood [8].

Despite the assumed importance of epigenetics in ovarian carcinogenesis, as in other malignancies, epigenetic aberrations that contribute to ovarian carcinogenesis or epigenetic mechanisms that regulate cancer-associated genes are less well understood than are genetic changes. Therefore, this review examines the current understanding of epigenetic mechanisms involved in the regulation of ovarian cancer-associated genes, including oncogenes and tumor suppressor genes, as well as epigenetic abnormalities that contribute to ovarian tumorigenesis. In addition, epigenetic therapies that target CICs, or drug resistant ovarian cancers, are discussed as promising approaches for the efficient treatment of ovarian cancer.

## 2. Epigenetic Changes in Cancer

It has long been known that cancers are caused by genetic alterations [9]. A number of genetic changes in oncogenes, including mutations, deletions, amplifications, rearrangements and translocations, as well as changes in tumor suppressor or microRNA (miRNA) genes, are involved in multistep carcinogenesis, and accumulate with tumor progression [10,11]. However, classical genetics alone cannot explain all the properties of cancer, and it is now understood that epigenetic abnormalities, in addition to genetic alterations, are involved in tumorigenesis [12]. Epigenetics is defined as heritable changes in gene expression that are not caused by DNA sequence alterations [12]. Epigenetic modifications include DNA methylation and histone modifications, which may differ between cancer and normal cells [11,12]. Recently, small non-coding miRNAs were reported to act as epigenetic regulators. Such miRNAs regulate gene expression through posttranscriptional silencing of target genes. Depending on the sequence complementarity between the miRNA and its target, miRNAs lead to cleave the mRNA or inhibit translation [13]. Changes in the expression of miRNAs during tumorigenesis were discovered in several cancers and they are associated with the prognosis and the progression of cancer in some cases.

DNA methylation is a well-known epigenetic mark. In humans and other mammals, DNA methylation occurs at cytosine residues in cytosine-guanine (CpG) dinucleotides and is controlled by enzymes called DNA methytransferases (DNMTs), including DNMT1, DNMT3A and DNMT3B. CpG sites are mainly distributed in CpG-rich regions, known as CpG islands. Most CpG islands are located in repetitive elements, such as centromeres, microsatellite sequences and the proximal promoter regions of approximately half of the genes in the genome of normal cells, where the islands are generally unmethylated [11].

The transcriptional inactivation of tumor suppressor genes by CpG island promoter DNA hypermethylation is well-known as one of the alterations that contributes to tumorigenesis in cancer cells [12]. At the same time, the genome of cancer cells undergoes global hypomethylation at repetitive sequences, or in tissue-specific or imprinted genes, whereas these regions are heavily hypermethylated in normal cells [14]. It is speculated that this global DNA hypomethylation contributes to tumorigenesis by causing chromosomal instability or the reactivation of transposable elements [12,14]. On the other hand, the first study showing the endogenous repeat element-driven activation of the oncogenic tyrosine kinase, CSF1R, was recently reported [15], suggesting that impaired epigenetic control and the subsequent transcriptional derepression of repeat elements play a role in tumorigenesis. This study further demonstrated that oncogenes can be activated by the derepression of endogenous repeats, in addition to genetic and epigenetic modifications. Loss of imprinting (LOI), which is involved in the development of tumors, is also associated with DNA hypomethylation [12]. Furthermore, it has also been suggested that the activation of normally silenced genes by promoter DNA hypomethylation is involved in tumorigenesis [14].

In addition to DNA methylation, histone modifications are epigenetic marks that are involved in chromatin structure and gene expression. The so-called “histone code” hypothesis posits that covalent modifications of histone tail residues act in concert to govern DNA packaging and thus regulate the access of transcriptional machinery to coding sequences [16,17]. Histone modifications occur at histone residues, such as lysine, arginine and serine (which can be methylated, acetylated and phosphorylated) and in particular, the histone methylation and acetylation status of specific lysine residues has been correlated with either active or repressive transcription [14,18]. Specifically, the trimethylation of histone H3 lysine 9 (H3K9me3), H3 lysine 27 (H3K27me3) and H4 lysine 20 (H4K20me3), in addition to H3K9 dimethylation (H3K9me2), facilitates transcriptional repression, whereas histone acetylation of histone H3 (H3Ac) and H4 (H4Ac), and the trimethylation of H3 lysine 4 (H3K4me3) are associated with transcriptional activation. The methylation of specific histone lysine residues is mediated by their cognate histone methyltransferases, and the recent discovery of histone lysine demethylases has indicated that the so-called “histone code” is highly signal-responsive and dynamic [18,19]. In embryonic stem cells, the “bivalent” colocalization of activating H3K4 methylation and the repressive H3K27 methylation of development-associated genes, followed by the lineage-specific loss of H3K4me3 or H3K27me3, has been reported to allow differentiated tissue silencing or expression [20,21]. Furthermore, a growing body of evidence suggests that gene expression may be regulated by interactions between multiple histone modifications or by crosstalk between DNA methylation and histone modifications [22–24]. In particular, histone H3K9 methylation and H3K27 methylation (mediated by the polycomb repressive complex 2 (PRC2) protein, EZH2) are linked to DNA methylation, as has been reported in several studies [22,25–27]. In fact, histone modifying enzymes such as histone deacetylase 1 (HDAC1) and HDAC2 were shown to interact with DNMT1 [28,29]. Moreover, heterochromatin protein, HP1, which binds H3K9 methylated histones, also cooperates with DNMT1 in mediating gene silencing [30]. EZH2, a component of PRC2, also binds directly to DNMTs [27] and the human polycomb-group protein (PcG), EED, was shown to interact with HDAC proteins [31]. Aberrant histone modifications, in addition to DNA methylation, are recognized as important epigenetic changes during tumorigenesis. While the promoters of tumor-suppressor genes are enriched with active histone marks in normal cells, the transcriptional silencing of those genes in cancer cells is reported to be associated with a loss of active histone marks, including H3K4me3 and histone acetylation, and a gain of repressive H3K9 methylations, H4K20me3 and H3K27me3 marks [14]. Moreover, the global loss of acetylation at histone H4K16 and the trimethylation of H4K20 were shown to be common features of human cancer cells [32].

Although not as well-established as the epigenetic regulators of DNA methylation and histone modifications, the recently discovered miRNAs are also thought to play a role in tumorigenesis by modulating tumor suppressor genes or oncogenes [13,33]. miRNAs might contribute to tumorigenesis by controlling various biological processes, including differentiation, proliferation, and apoptosis through regulation of or interactions with oncogenes or tumor suppressor genes. miRNAs can act either as an oncogene or tumor suppressor gene depending on their target genes [13,33].

Up-regulation of miRNAs that target tumor suppressor genes through overexpression, amplification, or epigenetic derepression might function as oncogenes by inhibiting the activity of an anti-oncogenic pathway. By contrast, the genetic mutation, deletion or epigenetic silencing of a tumor suppressor miRNA that normally represses expression of oncogenes might result in derepression of oncogenes, thereby gain of oncogenic function. The let-7 miRNAs, for example, which are down-regulated in lung cancer, negatively regulate the oncogenes *RAS* [34] and *HMGA2* [35], suggesting their possible role in the activation of oncogenes in lung cancer. In addition, the oncogene c-Myc was shown to be a target of let-7a in lymphoma cells [36], while miR-15a and miR-16-1 function as tumor suppressors by targeting *BCL2* in leukemia cells [37]. In contrast, miR-21 is up-regulated in several tumors and plays an oncogenic role by regulating the expression of the tumor suppressor *PTEN* in hepatocellular cancer [38]. miRNAs expression can also be transcriptionally activated or repressed through direct interaction with oncogene or tumor suppressor transcription factors. miR-34 was revealed to be induced by TP53 through direct binding of TP53 to the miR-34s, suggesting that TP53’s effects could be mediated in part by transcriptional activations of miRNAs [39–41]. c-myc was also shown to transactivate miRNAs such as miR-17–92 cluster [42], while it represses transcription of tumor suppressor miRNAs such as let-7 and miR-29 family members [43]. These data suggest that miRNAs play important roles in the oncogenic pathways through the regulation of multiple targets or mediation of oncogenic signals. Therefore, the discovery of key miRNAs that have multiple targets which are involved in different oncogenic pathways or that are mediators of oncogenic pathways might be important in the development of effective anti-cancer drugs. On the other hand, miRNAs can directly modulate epigenetic regulatory mechanisms by targeting enzymes responsible for DNA methylation (DNMT3A and DNMT3B) [44] and histone modifications (EZH2) [45].

In addition to the role of miRNAs in the development of tumors, miRNAs have been implicated in tumor progression by affecting adhesion, migration and invasion of cancer cells. miR-10b, a direct target of Twist 1, was up-regulated in metastatic breast cancer cells and ectopic miR-10b expression in non-metastatic breast cancer induced invasion and metastasis [46]. miR-373 and miR-520c were also shown to positively regulate cancer cell migration and invasion by blocking the adhesion molecule CD44 in breast cancer [47].

## 3. Epigenetic Inactivation of Tumor Suppressor Genes or Cancer-Associated Genes in Ovarian Cancer

It is well known that mutations in *TP53*, the most frequently mutated gene in cancer, result in loss-of-function, and that the loss of *BRCA1* or *BRCA2* function occurs through LOH or mutations [4]. It is also known that *PTEN* is inactivated by somatic mutations in ovarian cancer.

In addition to this genetic inactivation of tumor suppressor genes, epigenetic mechanisms also contribute to the inactivation or down-regulation of tumor suppressor genes or cancer-associated genes in ovarian cancer (Table 1). For example, *PTEN*, inactivated by LOH and mutation, is also down-regulated by promoter DNA methylation [48]. In addition, it was recently reported that deregulated miR-214 targeted the 3′ UTR region of *PTEN* resulting in the down-regulation of the PTEN protein expression [49]. These findings suggest that several epigenetic changes are involved in the down-regulation of *PTEN* in ovarian cancer. *BRCA1* inactivation in ovarian cancer has also been associated with promoter DNA methylation, in addition to mutation and LOH [50].

Loss of expression of the growth inhibitory imprinted genes, *DIRAS3 (ARH1)* and *PEG3*, also occurs through promoter DNA methylation, in addition to LOH [51]. Several other putative tumor suppressor genes including *RASSF1*, *DLEC1*, *CDKN2A*, *CDKN1A* and *MLH1* are also down-regulated by promoter methylation or histone modifications (Table 1). In particular, repressive histone methylation, H3K27me3, was shown to be responsible for *RASSF1* down-regulation in ovarian cancer cells [25]. In addition, repression of *ADAM19*, a SMAD target gene, has been associated with the repressive histone modifications, H3K27me3 and H3K9me2, and histone deacetylase in ovarian cancer cells, while the promoter of ADAM19 was unmethylated [52]. The decreased expression of GATA transcription factors (*GATA4* and *GATA6*) in ovarian cancer was found to correlate with the hypoacetylation of H3 and H4 and the loss of active H3K4me3. The genes could be reactivated by HDAC inhibitors, but not by DNA demethylating agents, suggesting that altered histone modifications, independent of DNA methylation, is one possible mechanism responsible for silencing the GATA transcription factors in ovarian carcinogenesis [53].

The aberrant expression of miRNAs in ovarian cancer compared to normal ovaries was found in several studies [49,54,55]. Among significantly deregulated miRNAs, miR-200a [49,54], miR-200c and miR-141 [54,55] were the most strongly up-regulated and their higher expression was significantly associated with poor prognosis in ovarian cancer [55]. Some among the up-regulated miRNAs were also shown to be capable of acting as oncogenes by repressing expression of tumor suppressor genes. For example, as mentioned above, miR-214 functions as an oncogene by targeting the tumor suppressor *PTEN* in ovarian cancer cells. It induced cell survival and cisplatin resistance through activation of Akt pathway by down-regulation of PTEN protein [49]. In addition, an increase in the expression of miR-200 family members correlated with the decreased expression of ZEB transcription factors, which are known to promote epithelial-mesenchymal transition (EMT) by repressing the expression of critical adhesion molecules of epithelial cells and miR-200 family targets ZEB1/2, suggesting that miR-200 family plays a role in ovarian tumor progression [56].

## 4. Epigenetic Derepression of Oncogenes or Cancer-Promoting Genes in Ovarian Cancer

It is generally known that oncogenes are activated or overexpressed by genetic alterations, including mutations or gene amplifications. In ovarian cancer, activating mutations in oncogenes such as *KRAS*, *BRAF* and *PIK3CA* are common in low-grade (type I) ovarian tumors, while *TP53* mutations are frequent in high-grade (type II) ovarian tumors [3]. However, a few oncogenes have been reported to be activated by spontaneous mutations in ovarian cancer, and gene amplifications alone cannot explain the activation or overexpression of oncogenes, although their frequency is higher than activating mutations [4]. These observations support the possibility that other mechanisms may be responsible for the activation of oncogenes in ovarian cancer.

**Figure 1.**
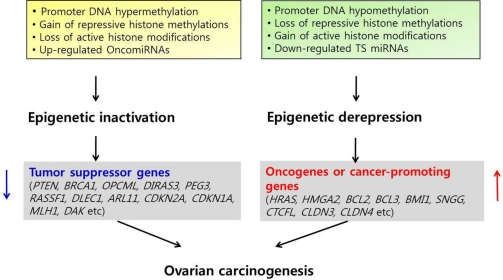
Epigenetic regulation of cancer-associated genes in ovarian cancer. In addition to the down-regulation of tumor suppressor genes by epigenetic inactivation, epigenetic derepression through DNA hypomethylation, loss of repressive histone modifications and gain of active histone modifications, together with down-regulated tumor suppressor (TS) miRNAs may contribute to ovarian carcinogenesis by the up-regulation of oncogenes or cancer-promoting genes. OncomiRNAs are miRNAs which act as oncogenes.

Unlike the well-known epigenetic silencing of tumor suppressor genes, the epigenetic activation of oncogenes or cancer-promoting genes is not well established. However, a growing number of recent findings support the theory that epigenetic mechanisms may promote cancer progression by the activation or overexpression of oncogenes or cancer-promoting genes in ovarian cancer as well as by the inactivation of tumor suppressor genes (Figure 1).

### 4.1. Activation of Oncogenes or Cancer-Promoting Genes by DNA Hypomethylation

In addition to global DNA hypomethylation, promoter DNA hypomethylation contributes to the activation of genes that are normally repressed in cancer cells. In ovarian cancer, it has been reported that the following cancer-promoting genes are overexpressed in association with promoter DNA hypomethylation: *SNGG* (synucelin-γ), encoding an activator of the MAPK and Elk-1 signaling cascades [77,78], *BORIS* (brother of the regulator of imprinted sites), a cancer-testis antigen gene and a paralog of *CTCF* [79] (Table 2). On the other hand, possible regulation of other cancer-germline (CG) or cancer-testis antigen genes by BORIS was recently investigated but it was shown that BORIS expression is not sufficient for induction of CG antigen gene expression and DNA hypomethylation in their promoter in ovarian cell lines, suggesting the involvement of additional mechanisms in the regulation of CG antigen expression in ovarian cancer [80]. Other cancer-associated genes including *MCJ* [81,82], *MAL* (mal, T-cell differentiation protein) [83], *HOXA10* [84] and *TUBB3* [85] are up-regulated in ovarian cancer in association with DNA hypomethylation.

### 4.2. Activation of Oncogenes or Cancer-Promoting Genes by Chromatin Modification

In addition to DNA hypomethylation, epigenetic derepression by histone modification is a possible mechanism underlying the overexpression of cancer-promoting genes, although DNA hypomethylation has been mainly reported as an epigenetic mechanism underlying the up-regulation of cancer-associated genes. Recently, we showed that the overexpression of claudin-3 and claudin-4, which promote ovarian cell invasion, is associated with epigenetic derepression through the loss of repressive histone modifications, suggesting that changes in histone modifications can also contribute to the activation of cancer-promoting genes, independently of DNA methylation [86].

### 4.3. Regulation of Oncogenes or Cancer-Promoting Genes by miRNAs in Ovarian Cancer

Deletion, mutation or epigenetic silencing of miRNAs can also lead to the overexpression of oncogenes or cancer-promoting genes [33]. An integrated approach to epithelial ovarian cancer found that miRNAs were down-regulated in advanced ovarian tumors by both genomic copy number loss (∼15%) and epigenetic silencing (>36%), and that down-regulated miRNAs contributed to transcriptional deregulation [87]. Among significantly down-regulated miRNAs in ovarian cancer, the most strongly down-regulated miRNAs include miR-125b [49,54,55] and the let-7 family [49,88].

With respect to the regulation of specific oncogenes or cancer-promoting genes by miRNAs in ovarian cancer, it was reported that miR-15a and miR-16 control oncogenic Bmi-1 expression as well as targeting anti-apoptotic Bcl-2 in ovarian cancer cell lines [89], while miR-9, was found to inhibit ovarian cancer growth by regulating NF-kB1 expression [90]. In addition, miR-125b targets proto-oncogenic BCL3, which can suppress ovarian cancer cell growth [91], and overexpressed ARID3B in ovarian cancer is a target of miR-125a [92]. Specifically, as ARID3B is involved in the mesenchymal phenotype development, ARID3B increase by repression of miR-125a is suggested to promote a mesenchymal morphology and contribute to ovarian cancer progression. It has also been mentioned that the overexpression of let-7i down-regulated oncogenic proteins such as H-RAS and HMGA2 to a significant extent in ovarian cancer cell lines [93]. These data support that deregulated miRNAs also might promote ovarian cancer progression by derepressing cancer-associated genes as well as by repressing the expression of the genes.

## 5. Epigenetics of Cancer-Initiating Cells in Ovarian Cancer

Recently, tumor cell heterogeneity has been explained by the existence of epigenetic variation in progenitor cells. This “epigenetic progenitor model” suggests that stem or progenitor cells become cancer cells following epigenetic changes, highlighting the crucial role of epigenetic alteration in cancer initiation [6].

Self-renewing CICs are thought to constitute a small population of cells responsible for tumor formation and maintenance within a tumor [97]. These CICs, first identified in leukemia [98], have been identified in several solid tumors including breast, brain, prostate, head and neck, colon, pancreas, lung, liver and melanoma [99]. Ovarian CICs were also identified and isolated in ovarian cancer patient ascites [100] and primary ovarian tumors [101,102]. Furthermore, several markers able to define a CIC population in primary ovarian tumors have been discovered, for example, CD44^+^CD117^+^ [101], CD133^+^ [103,104], CD24^+^ [104] and CD44^+^/MyD88^+^ [105]. Recent studies additionally reported that a phenotype of CD44^+^CD24^+^Epcam^+^ is enriched for ovarian CICs [106], and aldehyde dehydrogenase-1A1(ALDH1A1)-positive cells are a population with properties of ovarian CICs, which are associated with chemoresistance, and down-regulation of ALDH1A can resensitize chemotherapy in ovarian cancer [107].

Since the epigenetic modulation of gene expression is central to the maintenance of stem cell identity [108], CICs are also likely to be regulated by an epigenetic mechanism. Specifically, it has been suggested that bivalent modifications consisting of the coexistence of activating H3K4 methylation marks and repressive H3K27 methylation marks silence developmental genes in embryonic stem cells while keeping them poised for activation [20]. A bivalent chromatin pattern was also identified in pluripotent embryonic carcinoma cells as an epigenetic mechanism for gene regulation [109], supporting the association of chromatin modifications with CICs. It has also been suggested that miRNAs are involved in the dysregulation of CICs by modulating the pivotal signaling pathways of the “stem cell genes”, Notch, Hedgehog, Wnt/β-catenin, HMGA2, Bcl-2 and Bmi-1 [110]. Recently, the expression of CD133, which is one of the cell surface markers for CICs in several cancers, including ovarian cancer, was shown to be regulated by DNA methylation (an epigenetic modification) [103], supporting the hypothesis that the identity of CICs may be defined by epigenetic modulation of stem cellness-related genes.

## 6. Epigenetics and Drug Resistance in Ovarian Cancer

The standard chemotherapy strategy in treating ovarian cancer involves a combination of a platinum- (carboplatin or cisplatin) and a taxane- (paclitaxel or docetaxel) based therapy. Chemoresistance is a major problem that compromises the effects of the current chemotherapies in ovarian cancer, and it has been discovered that epigenetic changes in several genes are associated with chemoresistance in this type of cancer. Firstly, CpG DNA methylation of the *MLH1* mismatch repair genes was associated with a relapse of a chemoresistant ovarian tumor [111]. Subsequently, it was shown that the silencing of *SFRP5* (a Wnt antagonist) by DNA hypermethylation was related to platinum resistance of ovarian cancer [112]. High levels of methylation in the Methylation Controlled DNAJ (*MCJ*) gene resulted in loss of gene expression and correlated with a poor response to chemotherapy [82], while the epigenetic inactivation of *ASS1* (argininosuccinate synthetase) was also associated with resistance to platinum chemotherapy [113]. In addition to a loss of expression due to DNA methylation, it was shown that an increase in expression of the *MAL* (myelin and lymphocyte protein) gene is associated with DNA hypomethylation and platinum resistance [83]. Based on the association of DNA methylation of specific genes with platinum sensitivity, a global approach to the identification of biological pathways implicated in platinum chemoresistance in ovarian cancer cells was recently performed [114]. This study showed that the hypermethylation-mediated repression of cell adhesion and tight junction pathways and the hypomethylation-mediated activation of the cell growth-promoting pathways, PI3K/Akt, TGF-beta and cell cycle progression, might contribute to cisplatin resistance in ovarian cancer cells.

Several miRNAs and their predicted target mRNAs, which are associated with a response to chemotherapy in ovarian cancer cells, were recently identified [115]. It was reported that miR-214 induced cisplatin resistance by targeting *PTEN* [49], that *let-7i* was a down-regulated tumor suppressor gene in platinum-resistant ovarian tumors and that reduced *let-7i* expression increased resistance to cisplatin [93].

## 7. Epigenetic Therapy

Unlike genetic changes, epigenetic modifications, including DNA methylation and histone modifications, are reversible, which suggests that such modifications may be promising therapeutic targets. Therefore, it may be possible to reverse aberrant gene expression using epigenetic drugs that alter DNA methylation and histone modification patterns. Currently, several epigenetic compounds have been developed that target the DNA methylation and histone deacetylation enzymes, and these have either been approved for use or are being tested in clinical trials (Table 3).

**Figure 2.**
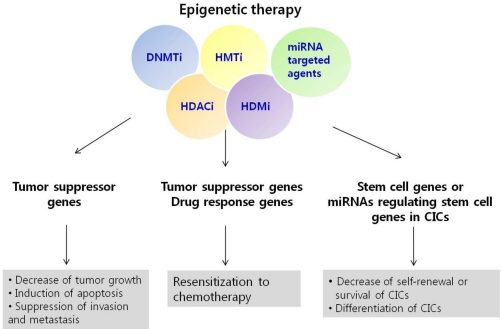
Strategies for epigenetic therapy in cancer treatment. The anti-tumor effects of DNA methyltransferase inhibitors (DNMTi) and histone deacetylase inhibitors (HDACi) have been demonstrated in preclinical studies and several of these inhibitors are under clinical trials for drug development in the treatment of cancer. Recently, a number of novel epigenetic therapies targeting histone methylation associated with the expression of cancer-associated genes, or miRNAs, have been suggested for cancer treatment. These epigenetic therapies are expected to show anti-tumor effects by inhibiting growth or inducing the apoptosis of tumor cells. Moreover, epigenetic therapies hold promise for resensitizing cancer cells to chemotherapy by modulating drug response genes or pathways. In addition to targeting tumor cells, targeting CICs by epigenetic therapy is also emerging as promising approach in the treatment of cancer. It is expected that the survival, or differentiation of CICs, could be regulated by epigenetic therapies.

The most commonly used DNA methyltransferase inhibitors (DNMTi) are nucleoside analogues, including 5-aza-cytidine (5-azaC), 5-aza-2′-deoxycytidine (5-aza-dC) and Zebularine [116–118]. These DNMTi are converted to deoxynucleotide triphosphates intracellularly, and may be incorporated into replicating DNA instead of cytosine, thereby trapping DNMT at the sites of the incorporated nucleosides. Importantly, these inhibitors cause the loss of DNA methylation and result in the re-expression of the tumor suppressor genes or the genes that were aberrantly silenced by DNA methylation in cancer cells, which can result ultimately in the inhibition of tumor cell growth or the induction of cell differentiation and cancer cell death [116] (Figure 2). Both 5-azaC and 5-aza-dC were recently approved by the U.S. Food and Drug Administration (FDA) for the treatment of myelodysplastic syndrome (MDS) [117].

In addition to DNA demethylation agents, several drugs that inhibit HDACs are under development as promising anti-cancer drugs because histone deacetylation is involved in suppressing critical genes such as tumor suppressor genes. HDAC inhibitors (HDACi) are reported to show anti-tumor effects by inhibiting cell growth, promoting apoptosis or inhibiting invasion or metastasis. These HDACi-induced effects are related to the induction of the cell cycle inhibitor p21, pro-apoptotic genes and E-cadherin, which suppress cell invasion and metastasis through promoter hyperacetylation of these genes [118,119] (Figure 2). The U.S. FDA recently approved Suberoylanilide Hydroxamic Acid (SAHA) for the treatment of T-cell cutaneous lymphoma, and several other HDACi are being developed and/or tested in clinical trials (Table 3).

Of recent interest is the development of drugs that target enzymes involved in histone methylation which also play a role in the silencing of tumor suppressor genes in cancer (Table 3). Recently, 3-Deazaneplanocin A (DZNep) was reported to deplete polycomb group proteins, inhibit repressive histone methylation including H3K27me3 and H4K20me3, and induce apoptosis in breast cancer cells [120]. A small molecule inhibitor was also shown to block H3K9me2 by inhibiting G9a histone methyltransferases [122]. On the other hand, polyamine [123] and oligoamine analogs [124] inhibit histone demethylases of H3K4, resulting in the up-regulation of aberrantly silenced genes.

There appears to be a level of synergy between DNA demethylation and histone acetylation in the re-expression of silenced genes in cancer [125] and a type of combination treatment is being tested in a clinical trial [126]. As the interactions between the various epigenetic modifications become clearer, further combination treatments of DNA and histone modification inhibitors may be investigated as promising approaches in the treatment of cancer. Recently, a combined epigenetic therapy, involving the histone methyltransferase EZH2 inhibitor and a HDAC inhibitor, was shown to be effective in acute myeloid leukemia (AML) cells [127]. In addition, the LSD1 inhibitor inhibited the growth of colon cancer when used in combination with a DNMT inhibitor [124]. Furthermore, these epigenetic agents can be used in combination with chemotherapy to augment anti-tumor effects. Epigenetic drugs can alleviate the resistance to other drugs, such as those used in chemotherapy, through the reactivation of DNA repair genes or other drug response genes (Figure 2) [128].

Therapeutic strategies for targeting the reactivation of epigenetically silenced tumor suppressor miRNAs or for targeting CICs that are refractory in standard chemotherapy are also promising (Figure 2) [129]. As a single miRNA can have multiple targets that are involved in different oncogenic pathways, modulating the level of a single miRNA might be effective in the treatment of cancer patients by affecting many pathways at the same time. Also, targeting a consistently deregulated miRNA group in several cancers might be a promising strategy in treating the cancer patients [13]. In addition, emerging evidence suggests that miRNAs are linked to the regulation of CICs [110], therefore, miRNA-targeted interventions may also be useful as anti-cancer therapies in addition to correcting the dysregulated expression of tumor suppressor genes or oncogenes in cancer. The miRNA antagonists could be used to inhibit oncogenic miRNAs, while miRNA mimics or lentiviral miRNAs have the potential to restore tumor suppressor miRNAs or to regulate stem cell genes. However, delivery of these miRNA-based therapeutics to the tumor remains a great challenge.

In addition to the reactivation of tumor suppressor genes or drug response genes, epigenetic drugs have been found to derepress immunogenicity-related genes, such as cancer/testis antigens, which are silent in normal cells but are expressed in various malignancies, suggesting that a combination of epigenetic therapy and immunotherapy might be useful [128].

However, there are several concerns in developing epigenetic therapies related to the non-specificity of these drugs. DNA hypomethylation agents might actually promote the development of some tumors, possibly by inducing chromosomal instability [130,131]. Simultaneous epigenetic derepression of oncogenes, or cancer-promoting genes, with the restoration of tumor suppressors by epigenetic agents, may be responsible for the poor efficacy or recurrence after epigenetic therapies. Therefore, an understanding of the global changes in gene expression that occur after epigenetic drug treatment is essential.

## 8. Epigenetic Therapy in Ovarian Cancer

With the advances in understanding of the molecular pathogenesis of ovarian cancer, several promising novel targets for therapy have been identified in epithelial ovarian cancer, and the agents targeting them are currently under development [2,5]. Among these therapeutic targets, several, including PI3K/Akt and Src signaling are known to be involved in chemotherapy resistance [2]. The recurrence of malignancy or the resistance to current therapies is one of the greatest concerns in the treatment of ovarian cancer. In particular, epigenetic inhibitors hold promise for overcoming chemoresistance in ovarian cancer through the restoration of drug response genes and pathways [8]. In fact, it was shown that the DNMT inhibitor, decitabine, decreased cisplatin resistance in both ovarian cancer cells and a mouse xenograft through demethylation of the hMLH1 promoter [132]. It was also demonstrated that the HDAC inhibitors, PXD101 (belinostat) and valproic acid, could resensitize chemotherapy resistant ovarian cancer cells [133,134]. Furthermore, a combination of decitabine and belinostat treatments showed greater cisplatin sensitization of a platinum-resistant mouse xenograft than either single treatment alone [135]. Based on the preclinical results of DNMT inhibitors or HDAC inhibitors, epigenetic drugs are undergoing clinical trial investigations for the treatment of recurrent resistant ovarian cancer [136]. Recently, two clinical trials [137,138] provided the first clinical evidences that DNA hypomethylation agent can reverse platinum resistance in ovarian cancer patients, suggesting the clinical benefits of epigenetic drugs in the treatment of chemoresistant or recurrent advanced cancer. In addition to DNMT inhibitors and HDAC inhibitors, several other epigenetic therapies, including inhibitors of histone methylation or histone demethylation, and miRNA targeting molecules could be used in the treatment of ovarian cancer in combination with chemotherapy or other therapies, although the anti-tumor effect of such therapies has not yet been proven.

On the downside, epigenetic drugs may simultaneously derepress cancer-promoting genes or oncogenes and restore tumor suppressor genes in ovarian cancer. Growing evidence shows that cancer-promoting genes or oncogenes are likely to be up-regulated by epigenetic mechanisms in ovarian cancer [8]. By combining the epigenetic treatment which up-regulates cancer-promoting genes or oncogenes with other therapies targeting those up-regulated genes, the augmentation of anti-tumor effect of epigenetic therapies can be expected. Therefore, it is very important to identify the molecular targets, for which targeting by epigenetic therapy together with other therapies causes a synthetic lethality. For example, the combined treatment of therapeutic antibodies targeting membrane proteins whose expression could be increased by epigenetic therapy in cancer cells (e.g., claudin-3 or claudin-4 in ovarian cancer cells) with epigenetic treatment could be a promising approach for the treatment of cancer. Consequently, a comprehensive understanding of the genes affected, together with a knowledge of the global genomic/epigenomic changes in ovarian cancer that occur after epigenetic drug treatment would be required to develop a more effective treatment strategy in combination with epigenetic therapy.

## 9. Conclusions

Given the high mortality rate for ovarian cancer patients due to difficulties in early detection and recurrence after current chemotherapy, the identification of promising therapeutic targets for molecular targeted therapy as well as the identification of relevant biomarkers for early detection is an immediate and crucial goal. Growing evidence supports the importance of epigenetic changes in tumorigenesis as much as the classical, and better known, genetic changes. Moreover, the crucial role of epigenetic aberrations during the early stages of tumorigenesis, and their link to CICs suggests that epigenetically dysregulated genes or pathways in cancer cells may be promising targets for the prevention or treatment of cancer. Several cancer-associated genes that play a role in the developmental progression of ovarian cancer are also epigenetically regulated. Even though it is not well established that oncogenes or cancer-promoting genes can be activated by epigenetic mechanisms, a few recent studies suggest that several epigenetic modifications, in addition to DNA hypomethylation are also likely to be involved in the activation or overexpression of cancer-associated genes. In particular, understanding the epigenetic derepression of oncogenes, or cancer-promoting genes, would be important for the development of epigenetic-based therapies used in combination with other therapies. With the development of high-throughput genomic/epigenomic approaches, it is now possible to identify the global differences between cancers and normal tissues. Thus, a comprehensive understanding of both the genomics and epigenomics of ovarian cancer through the use of integrated approaches should make it possible to identify epigenetically activated oncogenes or ovarian cancer-promoting genes. This will facilitate the identification of more significant biomarkers and promising novel therapeutic targets for ovarian cancer, and consequently, will contribute to the improved detection of ovarian cancer and its treatment.

## Figures and Tables

**Table 1.** Epigenetically down-regulated genes in ovarian cancer.

**Gene Symbol**	**Gene name**	**Chromosome**	**Mechanism of Down-Regulation**	**Reference**
**Tumor Suppressor Genes Down-Regulated by Both Genetic and Epigenetic Changes**				
*PTEN*	Phosphatase and tensin homolog	10q23	LOH, mutation	[4]
			Promoter DNA methylation miRNA (miR-214)	[48]
				[49]
*BRCA1*	Breast cancer 1, early onset	17q21	Mutation, LOH	[4]
			Promoter DNA methylation	[50]
*OPCML*	Opioid binding protein/cell adhesion molecule-like	11q25	LOH, mutation	[4]
			Promoter DNA methylation	[57]
*DIRAS3 (ARHI)*	DIRAS family, GTP-binding RAS-like 3	1p31	Imprinting, LOH, promoter DNA methylation	[51,58]
			Transcription down-regulated by E2F1 and E2F4	[4]
*PEG3*	Paternally expressed 3	19q13	Imprinting, LOH, promoter DNA methylation	[51]
TES *	Testis-derived transcript (3 LIM domains)	7q31.2	LOH, promoter DNA methylation	[59,60]
MYO18B *	Myosin XVIIIB	22q12.1	Mutation, promoter DNA methylation	[61]

**Tumor Suppressor Genes Down-Regulated By Epigenetic Changes**				
*RASSF1*	Ras association	3p21	Promoter DNA methylation	[62]
*(RASSF1A)*	(RalGDS/AF-6) domain family member 1		Histone methylation (H3K27me3)	[25]
*DLEC1*	Deleted in lung and esophageal cancer 1	3p22.3	Promoter DNA methylation, histone hypoacetylation	[63]
*ARL11 (ARLTS1)*	ADP-ribosylation factor-like 11	13q.14	Promoter DNA methylation	[64]
*CDKN2A(p16)*	Cyclin-dependent kinase inhibitor 2A (melanoma, p16, inhibits CDK4)	9p21	Promoter DNA methylation	[65,66]
*CDKN1A(p21)*	Cyclin-dependent kinase inhibitor 1A (p21, Cip1)	6p21.2	Hypoacetylation of H3Ac and H4Ac	[67]
*MLH1(hMLH1)*	MutL homolog 1, colon cancer, nonpolyposis type 2 (*E. coli*)	3p21.3	Promoter DNA methylation	[68,69]
*DAK(DAK1)*	Death-associated protein kinase 1	11q12.2	Promoter DNA methylation	[70]
*CDH1(E-cadherin)*	Cadherin 1, type 1, E-cadherin (epithelial)	16q22.1	Promoter DNA methylation	[71]
*FBXO32*	F-box protein 32	8q24.13	Promoter DNA methylation	[72]
CTGF *	Connective tissue growth factor	6q23.1	Promoter DNA methylation	[73]
ANGPTL2 *	Angiopoietin-like protein 2	9q33.3	Promoter DNA methylation	[74]

**Cancer-Associated Genes Down-Regulated By Epigenetic Changes**				
*ICAM1*	Intercellular adhesion molecule 1	19p13.3–p13.2	Promoter DNA methylation	[75]
*PCSK6 (PACE4)*	Proprotein convertase subtilisin/kexin type 6	15q26.3	Promoter DNA methylation and histone deacetylation	[76]
*GATA4*, *GATA6*	GATA binding protein 4 GATA binding protein 6	8p23.1 p22 18q11.1 q11.2	Hypoacetylation of H3Ac and H4Ac, loss of H3K4me3	[53]
*ADAM19*	ADAM metallopeptidase domain 19	5q33.3	Repressive histone modifications (H3K27me3 and H3K9me2)	[52]
*ZEB1*	Zinc finger E-box binding homeobox 1	10p11.2	miRNA (miR-200 family)	[56]
*ZEB2*	Zinc finger E-box binding homeobox 2	2q22.3	miRNA (miR-200 family)	[56]

* putative or candidate tumor suppressor genes. LOH, loss of heterozygosity.

**Table 2.** Epigenetically up-regulated genes in ovarian cancer.

**Gene Symbol**	**Name**	**Chromosome**	**Mechanism of Up-Regulation**	**Reference**
**Oncogene or Proto-Oncogenes**				
*HRAS*	v-Ha-ras Harvey rat sarcoma viral oncogene homolog	11p15.5	miRNA (Let-7i)	[93]
*HMGA2*	High mobility group AT-hook 2	12q15	miRNA (Let-7i)	[93]
*BCL2*	B-cell CLL/lymphoma 2	18q21.3	miRNA (miR-15a and miR-16)	[89]
*BCL3*	B-cell CLL/lymphoma 3	19q13.1–q13.2	miRNA (miR-125b)	[91]
*BMI1*	BMI1 polycomb ring finger oncogene	10p11.23	miRNA (miR-15a and miR-16)	[89]
*NFKB1(NF-kappa B1)*	Nuclear factor of kappa light polypeptide gene enhancer in B-cells 1	4q24	miRNA (miR-9)	[90]

**Cancer-Promoting Genes**				
*SNCG*	Synuclein, gamma (breast cancer-specific protein 1)	10q23.2–q23.3	Promoter DNA hypomethylation	[77,78]
*CTCFL(BORIS)*	CCCTC-binding factor (zinc finger protein)-like	20q13.31	Promoter DNA hypomethylation	[79]
*CLDN3*	Claudin-3	7q11.23	Promoter DNA hypomethylation and histone acetylation	[94]
			Loss of repressive histone methylations	[86]
*CLDN4*	Claudin-4	7q11.23	Promoter DNA hypomethylation, histone acetylation	[95,96]
			Loss of repressive histone methylations	[86]

**Cancer-Associated Genes**				
*DNAJC15(MCJ)*	DnaJ (Hsp40) homolog, subfamily C, member 15	13q14.1	Promoter DNA hypomethylation	[81,82]
*MAL*	Mal, T-cell differentiation protein	2cen-q13	Promoter DNA hypomethylation	[83]
*HOXA10*	Homeobox A10	7p15.2	Promoter DNA hypomethylation	[84]
*TUBB3*	Class III ß-tubulin	16q24.3	Promoter DNA hypomethylation and histone acetylation	[85]
*ARID3B*	AT-rich interactive domain 3B	15q24	miRNA (miR-125a)	[92]

**Table 3.** Epigenetic therapeutic compounds, approved or under development.

**Epigenetic Drugs**		**Compound (Commercial name)**	**Target**	**Status**	**Indication**	**Reference**
DNA methylation Inhibitor (DNMTi)	Nucleoside analog	5-aza-cytidine (Vidaza)	DNMT	FDA approved	MDS	[116–118]

		5-aza-2′-deoxy cytidine (decitabine)	DNMT	FDA approved	MDS	

		Zebularine	DNMT			

	Non-nucleoside	Hydralazine	DNMT	Phase I		

HDAC inhibitor (HDACi)	Hydroxamate	SAHA (Vorinostat)	Class I, II HDACs	FDA approved	T cell cutaenous lymphoma	[116,118,119]

		TSA (Tricostatin A)	Class I, II HDACs	Preclinical		

		LBH589 (Panobinostat)	Class I, II HDACs	Phase I/II		

		PXD101 (Belinostat)	Class I, II HDACs	Phase I/II		

		PCI-24781	Class I, II HDACs	Phase I		

	Aliphatic acid	Sodium phenyl butyrate	Class I, IIa HDACs	Phase I/II		
		Valproic acid	Class I, IIa HDACs	Phase I/II		

	Cyclic peptide	FK228 (Romidepsin)	HDAC1, 2	Phase I/II		

	Benzamide	MGCD0103	Class I	Phase I/II		

Histone methyltrans ferase inhibitors (HMTi)	S-adenosylhomocysteine hydrolase inhibitor	3-Deazaneplanocin A (DZNep)	Polycomb group proteins			[116,117,120]

	Fungal mycotoxin	Chaetocin	SU(VAR)3-9			[121]

	Small molecule inhibitor	BIX-01294	G9a histone methyl transferase			[122]

Histone demethylase inhibitor (HDMi)	Polyamine analog	Polyamine analog	Histone demethylase LSD1			[123,124]
